# The protective effects of Saudi propolis against hepatic injury induced by gold nanoparticles in adult male albino rats

**DOI:** 10.14202/vetworld.2025.252-262

**Published:** 2025-02-08

**Authors:** Sarah A. Althubyani

**Affiliations:** Department of Biology. College of Science. Taibah University, Madinah, Saudi Arabia

**Keywords:** antioxidants, gold nanoparticles, hepatotoxicity, lipid metabolism, oxidative stress, rats, Saudi propolis

## Abstract

**Background and Aim:**

Gold nanoparticles (GNPs) are widely used in industrial and medical applications due to their unique properties but may induce oxidative stress and hepatotoxicity. Propolis, a bee-derived natural product with potent antioxidant and anti-inflammatory properties, shows promise as a hepatoprotective agent. This study evaluates the protective effects of Saudi propolis against GNP-induced hepatic damage by examining oxidative stress, lipid metabolism, and liver function. This study aimed to investigate the hepatoprotective effects of Saudi propolis against oxidative damage and lipid dysregulation induced by GNPs in male albino rats.

**Materials and Methods:**

A total of 180 adult male rats were divided into six groups: (1) Control (saline), (2) Propolis (100 mg/kg), (3) GNPs (10 nm, 0.2 mg/kg/day), (4) GNPs (30 nm, 0.2 mg/kg/day), (5) GNPs (10 nm) + propolis, and (6) GNPs (30 nm) + propolis. Treatments were administered daily for 5, 10, or 15 days. Blood and liver samples were analyzed for oxidative stress markers, liver enzymes (aspartate transaminase, alanine transaminase, alkaline phosphatase, and glutamyl transpeptidase), lipid peroxidation (malondialdehyde [MDA]), antioxidant enzymes (superoxide dismutase [SOD] and glutathione peroxides [GPx]), and lipid profiles (cholesterol [CHO] and triglyceride [TG]).

**Results:**

Rats treated with GNPs showed elevated liver enzymes, lipid peroxidation, and oxidative stress, accompanied by increased CHO and TG levels. In contrast, co-administration of Saudi propolis significantly mitigated these effects, restoring MDA, SOD, and GPx levels close to control values. The hepatoprotective effects were more pronounced for 10 nm GNPs than 30 nm. After 15 days, TG levels returned to near-normal levels, while CHO levels improved but remained elevated.

**Conclusion:**

Saudi propolis exhibits significant protective effects against GNP-induced hepatic damage, primarily due to its antioxidant properties and ability to reduce oxidative stress and lipid peroxidation. The findings provide evidence for the therapeutic potential of propolis in managing nanoparticle-induced liver toxicity.

## INTRODUCTION

Gold nanoparticles (GNPs) have recently garnered much attention due to their large surface area, high biocompatibility, and stability [1], making them highly effective for use in the healthcare industry in drug delivery, biomedical imaging, and catalysis [2]. However, despite great interest in their biomedical application, only a few clinical trials of GNP drugs have been approved by the US. Food and Drug Administration [3] as they exhibit cytotoxicity, genotoxicity, and immunotoxicity, as well as increased reactivity in biological systems [4]. In animals, the liver is the primary organ metabolizing medicines, micro-organisms, and other environmental factors [5]. Most hepatotoxic drugs cause oxidative stress, which is a major contributing factor to liver injury. They do this by either directly harming cells or interacting with inflammation to cause cell death [6]. When harmful substances are metabolized, reactive oxygen species (ROS) are produced that damage cells, damage mitochondria, and cause apoptosis. Activated Kupffer cell-induced inflammation intensifies tissue damage, whereas protein misfolding-induced chronic ER stress results in cell death, eventually contributing to reduced liver function [7]. The most significant indicators of the potential hepatotoxicity of mineral nanoparticles include gamma-glutamyl transpeptidase (γGT), aspartate transaminase (AST), alanine transaminase (ALT), alkaline phosphatase (ALP), and bilirubin. These substances are generally tested in plasma or serum, and liver injury typically increases their concentrations [8]. Saudi propolis, a waxy material obtained from various plant exudates [9], has a long history of use for mouthwash, wound prevention, mummification, and other purposes [10]. Plants use various phytochemicals to protect themselves against external pathogens [11]. Many of these compounds have been isolated and are used to treat various illnesses while providing excellent sources of vitamins and antioxidants [10]. Saudi propolis is a natural source of antioxidants and restores cellular function by preventing several side effects typically caused by alien substances in the body [12].

Saudi propolis contains anti-inflammatory, antibacterial, antiviral, and antioxidant properties [13]. Many hepatic conditions, such as acute liver failure, alcoholic and nonalcoholic fatty liver diseases, liver fibrosis, liver cirrhosis, and liver cancer, can be successfully treated with propolis [12].

This study aimed to determine the extent to which propolis mitigates the adverse effects of GNPs on liver function, lipid metabolism, and oxidative stress. The outcomes highlight the antioxidant and hepatoprotective potential of Saudi propolis in the context of nanoparticle-induced toxicity. This study provides unique insight into the protective effects of Saudi propolis against liver damage caused by GNPs. Unlikeearlier studies [10, 13], this study incorporated extensive biochemical evaluations of liver activity, oxidative stress indicators, and lipid profiles, providing fresh information on the potential reduction of oxidative stress and hepatotoxicity associated with nanomaterials. Our results provide a new perspective on Saudi propolis as a natural remedy for nanoparticle-induced liver damage.

## MATERIALS AND METHODS

### Ethical approval

All procedures were conducted with strict adherence to ethical guidelines and were approved by the Research Ethics Committee of the College of Pharmacy, Taibah University (COPTU-REC) (Ref. No. coptu-rec-89-20240410). Live animal models were essential for this research, as alternative *in vitro* methods could not replicate the systemic physiological responses required to meet the study objectives. In this study, animal care was performed following Directive 2010/63/EU of the European Parliament and of the Council of 22 September 2010 and national rules (Parliament, European and Council, European, 2010).

### Study period and location

**Figure 1:**
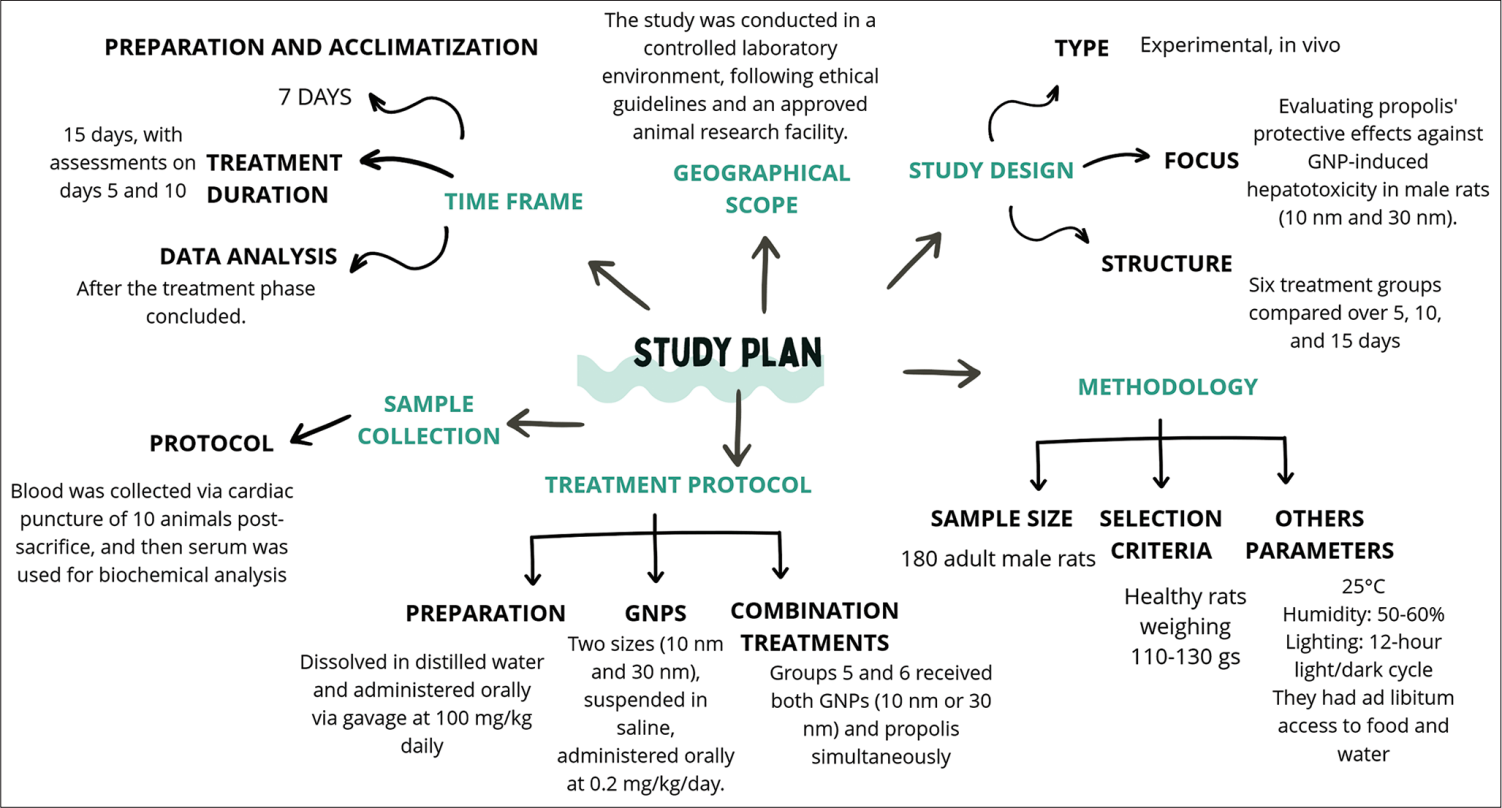
Plan for the study area.

The study was conducted from May 2024 to June 2024 at the Department of Biology, College of Science, Taibah University, Madinah, Saudi Arabia. The study plan is shown in Figure 1.

### Chemicals

Two sizes of GNPs were obtained from nanoComposix (San Diego, CA, USA) (https://nanocomposix.com/): 10-nm lot no. DAC1308 and 30-nm lot no. IXW0102. Saudi Propolis was obtained from the Tower of Flowers Trading Company (Saudi Arabia). AST, ALT, ALP, and gammaglutamyl transpeptidase (γGT) assay kits were procured from the Creative Approach to Bioscience, Germany (https://spectrum-diagnostics.com): (catalog No. 259 001, 263 001, 215 001, and 247001, respectively). *Superoxide dismutase* (SOD), glutathione peroxides (GPx), and *malondialdehyde* (MDA) were measured using enzymatic colorimetric methods using Biodiagnostic kits, Egypt (https://biodiagnostics.co.uk/divisions/autoimmune-disease/diagnostics-kits-reagents/) (catalog No. SD 25 21, GP25 24 and MD 25 29, respectively).

cholesterol (CHO) and triglyceride (TG) were measured using a spectrum diagnostics reagent (Spinreact, S.A./S.A.U Ctra., Spain (https://www.spinreact.com/en/home.html#accept) (catalog No. SP41021 and MX41031).

### Preparation of aqueous Saudi propolis extract

Saudi propolis was obtained from the Tower of Flowers Trading Company (Saudi Arabia). Propolis was collected from the southern region of Saudi Arabia. Bees feed on the nectar of trees and flowers in apiaries. Using ethanol extracts of Saudi propolis, as described by Paviani *et al*. [14], Saudi propolis extraction was performed using 250 g of propolis powder, which was transferred to a 1000 mL volumetric flask. The process was then continued to 1000 mL using 70% ethanol high-performance liquid chromatography grade without bright light and moderately stirred using a magnet stirrer for 1 day at room temperature (25°C). After a week, the extracts were passed through a filtration pump to remove the solvent, which was then evaporated in a vacuum oven at 60°C to produce pure Saudi propolis extracts, which were then diluted with saline and administered to rats. A chromatographic separation gaseous apparatus (Model 6980, Palo Alto Company, California, USA) (https://www.paloaltonetworks.com/) was used to identify the components of Saudi propolis.

### Propolis sample analysis

The components of the Saudi propolis sample were analyzed using gas chromatography-mass spectrometry (GC-MS). The sample was prepared by dissolving propolis in ethanol and filtering it to remove impurities. The analysis was conducted on a chromatographic separation gaseous equipment (Model 6980 made by Palo Alto Company, California, USA) (https://www.paloaltonetworks.com/) to identify the components of Saudi propolis.

### Experimental animals

In total, 180 adult male albino rats (*Rattus rattus*) of age 5–6 weeks (weight 110–130 g) in good health were employed. Only male rats were selected for the study to minimize potential hormonal variations, such as those associated with the estrous cycle in females, which could influence biochemical and oxidative stress parameters.

The acclimation period of 7 days was adopted and the animals were fed a regular pellet diet and were watered. The diet comprised approximately 20% protein, 5% fiber, 3.5% fat, 6.5% ash, and vitamin mixes. Water and food were made available throughout the experiment. The rats were kept at 25°C with a 12:12 light/dark cycle in appropriate cages with 10 rats/cage. The number of rats used in the study was determined through statistical power analysis to ensure reliable and robust results. The study design includedsix experimental groups, each tested at three different time points, necessitating a larger sample size to achieve comprehensive and statistically significant outcomes.

### Study design and experimental procedure

The rats were randomly divided into six groups, each containing 30 rats (three replicates of 10 rats in each group), as listed in Table 1.

**Table 1:** Study design and experimental procedure.

Groups	Dose	Days	No. of rats	Size of the nanoparticles
Group 1 (control)	Saline (orally)	5, 10, and 15 days	10 in each period	-
Group 2	100 mg/kg of propolis	5, 10, and 15 days	10 in each period	-
Group 3	0.2 mg/kg/day of oral GNP solution	5, 10, and 15 days	10 in each period	10 nm
Group 4	0.2 mg/kg/day of oral GNP solution	5, 10, and 15 days	10 in each period	30 m
Group 5	0.2 mg/kg/day of GNP solution+100 mg/kg of propolis	5, 10, and 15 days	10 in each period	10 nm
Group 6	0.2 mg/kg/day of GNP solution+100 mg/kg of propolis	5, 10, and 15 days	10 in each period	30 m

GNP=Gold nanoparticles

### Biochemical analysis

Blood samples from each group were drawn on day 7 from the heart in sterile, clean, and labeled tubes after the trial. The tubes were classified as follows:

A. Blood collection tube without using an anticoagulant. The blood was clotted at 25°C for 30 min and it was centrifuged at 4°C for 15 min at 4,000 rpm. After acquisition, serum samples were split into small quantities and stored at −80°C in a deep freezer for examination using a spectrophotometer (PD-303UV).

• The analysis included liver enzymes (AST, ALT, ALP, and γGT) according to the manufacturer’s protocols (https://spectrum-diagnostics.com/enzymes/). The working reagent and sample were mixed, and the initial absorbance was read after 60 s at the wavelength specified by the protocol. It was read again after 1, 2, and 3 min. The mean absorbance change per minute (ΔA/min) was determined.

• Lipid peroxide (MDA), the working reagent, was mixed well, heated for 30 min, and then the sample was added; the absorption of the sample was read against blank and standard against distilled water at 534 nm.

• Blood lipid analysis (CHO and TG) was performed according to the manufacturer’s protocol (https://www.spinreact.com/en/home.html). Briefly, the sample was incubated with the working reagent for 5 min at 37°C, and the absorption of the sample was read against a blank at 505 nm.

B. A blood collection tube with an anticoagulant (ethylene diamine tetra acetic acid) was used for the assay of SOD and Gpx in erythrocyte lysates according to the manufacturer’s protocols (https://biodiagnostics.co.uk/divisions/autoimmune-disease/diagnostics-kits-reagents/). The erythrocytes were washed four times with 3 mL of 0.9% sodium chloride solution. The washed centrifuged erythrocytes were made up to 2.0 mL with cold redistilled water, mixed, and left to stand at +4 **°**C for 15 min. The working reagent and sample were mixed, and the decrease in absorbance at 340 nm/min was recorded. (A_340_/min) for 3 min against deionized water.

### Statistical analysis

Statistical analyses were conducted using Statistical Package for the Social Sciences (SPSS) Statistics version 8 (SPSS Inc., USA), focusing on a one-way analysis of variance and *post hoc* least significant difference tests for group comparisons. Data were presented as mean ± standard deviation, and statistical significance was determined at p < 0.05.

A sample size of 10 per group was chosen to ensure statistical robustness. Multiple time points (5, 10, and 15 days) were included in the study to evaluate temporal effects of treatments. The analysis was conducted separately for each time point to account for variations over time.

Enzyme activities (AST, ALT, ALP, and GT) and oxidative stress markers (MDA, SOD, and GPx) were analyzed using standard protocols. Lipid profiles (CHO and TG) were analyzed using spectrophotometric absorbance data, as described in the methods section.

Group-wise and temporal comparisons were performed to assess treatment effects, focusing on differences between control, GNP-treated, and GNP + propolis groups, as well as the effects of nanoparticle size (10 nm vs. 30 nm).

Trend analyses over the three-time points were conducted to observe temporal changes in parameters, emphasizing their biological relevance. Tables were produced in Microsoft Excel 2021 (Microsoft Corporation, Washington, USA).

## RESULTS

**Figure 2:**
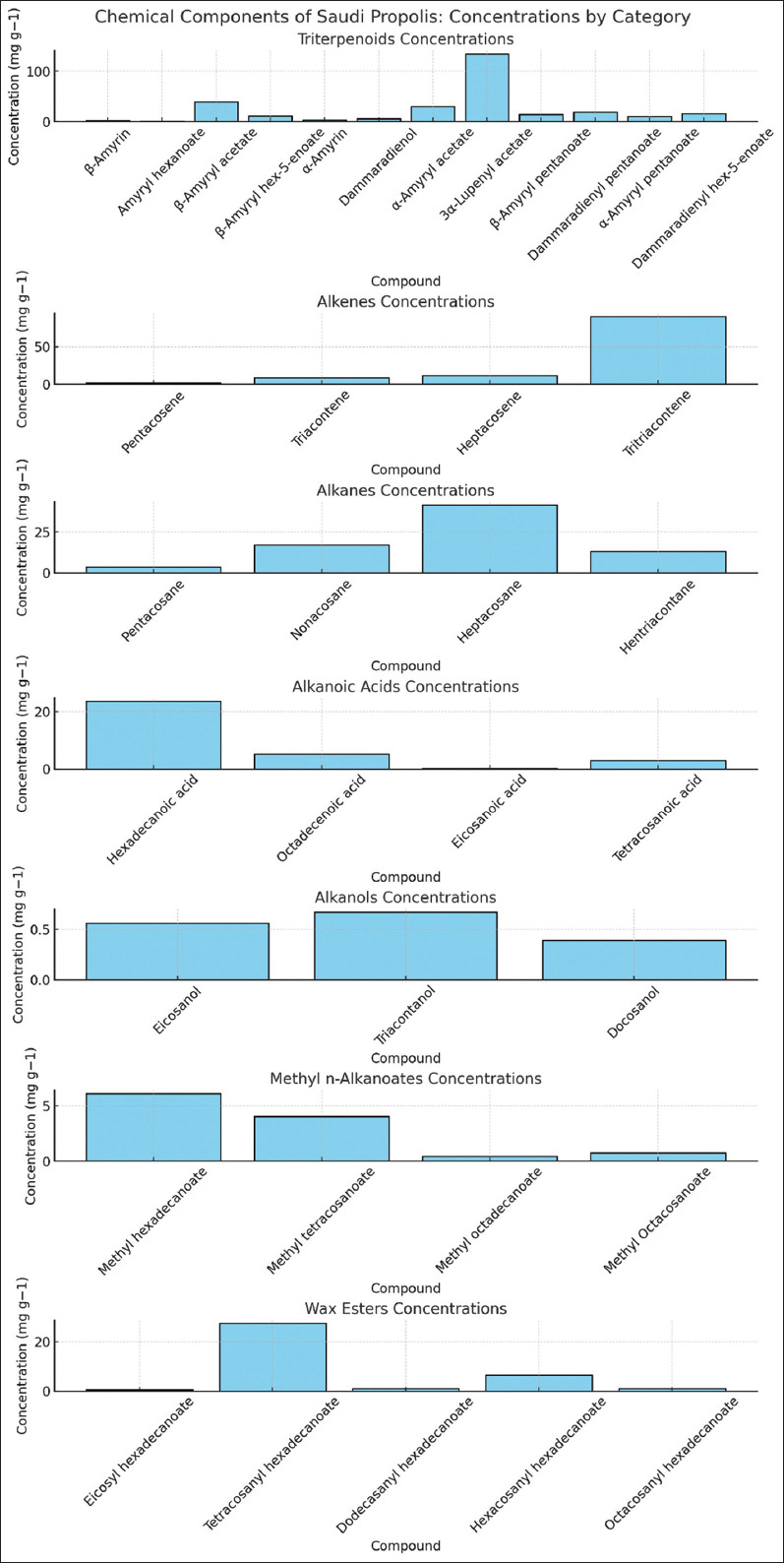
Chemical profile of Saudi propolis.

The components of the Saudi propolis sample were separated using the gas chromatographic separation equipment GC-MS and the sample was prepared by dissolving propolis in ethanol and filtering it to remove impurities.The findings indicated that 38 compounds were identified in propolis, as shown in Figure 2 and Table S1. Triterpenoids, alkenes, alkanes, alkanoic acids, alkanol, methyl n-alkanoates, and wax esters were the main compounds.

**Table S1:** Chemical components of Saudi propolis.

Compound	Concentrations (mg g^−1^)	Compound	Concentrations (mg g^−1^)
Triterpenoids			
β -Amyrin	2.29	Amyryl hexanoate	1.20
β -Amyryl acetate	39.84	β -Amyryl hex-5-enoate	11.22
α-Amyrin	3.08	Dammaradienol	5.34
α-Amyryl acetate	30.12	3α-Lupenyl acetate	134.45
β -Amyryl pentanoate	14.04	Dammaradienyl pentanoate	18.54
α-Amyryl pentanoate	10.52	Dammaradienyl hex-5-enoate	15.61
3β -Lupenyl acetate	12.7	α -Amyryl hex-5-enoate	4.50
Alkenes			
Pentacosene	1.70	Triacontene	8.22
Heptacosene	11.32	Tritriacontene	90.34
Alkanes			
Pentacosane	3.47	Nonacosane	17
Heptacosane	41.48	Hentriacontane	13.06
Alkanoic Acids			
Hexadecanoic acid	23.56	Eicosanoic acid	0.17
Octadecenoic acid	5.22	Tetracosanoic acid	2.92
Alkanols			
Eicosanol	0.56	Triacontanol	0.67
Docosanol	0.39		
Methyl n-Alkanoates			
Methyl hexadecanoate	6.12	Methyl tetracosanoate	4.05
Methyl octadecanoate	0.43	Methyl Octacosanoate	0.76
Wax Esters			
Eicosyl hexadecanoate	0.45	Tetracosanyl hexadecanoate	27.56
Dodecasanyl hexadecanoate	0.89	Hexacosanyl hexadecanoate	6.45
Octacosanyl hexadecanoate	0.98		

**Figure 3:**
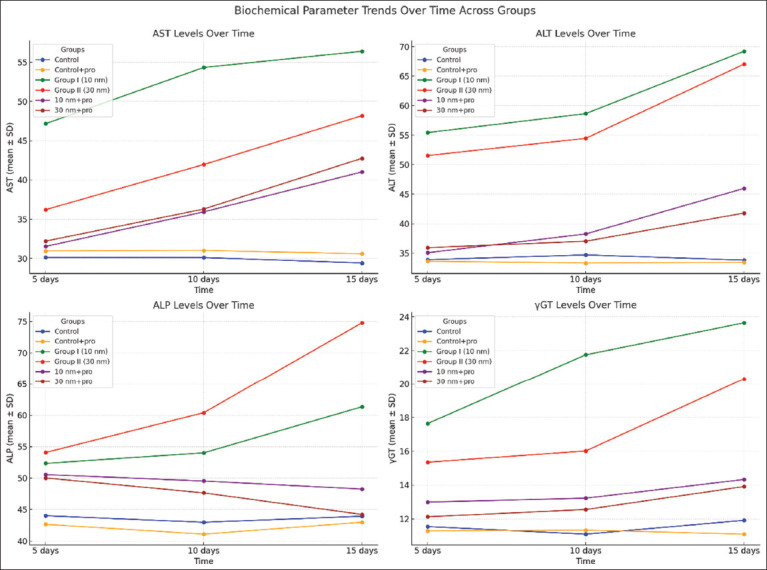
Trend of liver enzyme (AST, ALT, AMP, and γGT) levels throughout treatment. The graphs show significant changes among all experimental groups over 5, 10, and 15 days. AST=Aspartate transaminase, ALT=Alanine transaminase, AMP=Adenosine monophosphate, γGT=Gamma-glutamyl transpeptidase.

The results of the enzyme activities of AST, ALT, ALP, and γGT in male rats are summarized in Figure 3 and Table 2. Male rats exposed to GNPs (0.2 mg/kg/day) showed an increase in the serum enzyme activities of ALT, AST, ALP, and γGT levels comparable to control. In addition, the Saudi propolis-treated group (100 mg/kg/day) did not show any changes compared with the control. Combination treatment (GNPs with propolis) significantly decreased enzymatic activity compared with GNP alone. The same observations were recorded for GNPs of two sizes at different periods. The administration of GNPs (10 and 30 nm) with propolis for 5 days resulted in a highly significant decrease in AST and ALT levels compared with the GNP-treated groups. It is almost identical to the control. In contrast to the GNP-treated groups, there was a highly significant reduction in ALP levels in the 30 nm GNP-treated group for 15 days. No discernible difference from the control was observed. Compared with the GNP groups, there was a substantial drop in serum γGT after propolis administration.

**Table 2:** Serum level of serum level of AST, ALT, AMP, and γGT (U/L) in different experimental groups at 5, 10, and 15 days (Mean ± SD).

Groups	5 days				10 days				15 days			
		
	AST	ALT	ALP	γGT	AST	ALT	ALP	γGT	AST	ALT	ALP	γGT
Group 1 (Control)	30.15 ± 1.43	33.90 ± 1.42	44.02 ± 1.02	11.54 ± 0.98	30.12 ± 1.53	34.72 ± 1.54	42.98 ± 0.88	11.09 ± 0.17	29.43 ± 1.45	33.82 ± 0.45	43.94 ± 1.07	11.91 ± 0.76
Group 2	30.96 ± 2.47	33.63 ± 0.85	42.66 ± 0.63	11.28 ± 0.29	31.02 ± 0.85	33.34 ± 0.43	41.09 ± 0.36	11.33 ± 0.22	30.61 ± 0.74	33.43 ± 0.93	42.99 ± 0.81	11.09 ± 0.58
Group 3	47.17 ± 1.32^a^	55.43 ± 1.54^a^	52.34 ± 1.76 ^a^	17.64 ± 0.56^a^	54.33 ± 2.12^a^	58.65 ± 2.02^a^	54.02 ± 2.08 ^a^	21.75 ± 1.43^a^	56.39 ± 1.56^a^	69.21 ± 1.05^a^	61.34 ± 1.87^a^	23.65 ± 1.32^a^
Group 4	36.22 ± 1.23^a^	51.54 ± 0.39^a^	54.07 ± 1.42^a^	15.35 ± 0.43^a^	41.97 ± 1.75^a^	54.46 ± 1.23^a^	60.40 ± 1.21^a^	16.02 ± 1.23^a^	48.19 ± 1.87^a^	67.03 ± 1.89^a^	74.80 ± 2.05^a^	20.30 ± 1.43^a^
Group 5	31.54 ± 1.47^b^	35.08 ± 0.33^b^	50.56 ± 0.38^a,b^	12.99 ± 0.37^a,b^	35.94 ± 1.04^a,b^	38.26 ± 0.54^a,b^	49.54 ± 0.37^a,b^	13.22 ± 0.42^a,b^	41.03 ± 1.31^a,b^	45.97 ± 1.49^a,b^	48.26 ± 2.18^a,b^	14.33 ± 0.79^a,b^
Group 6	32.22 ± 0.68^c^	35.93 ± 1.37^c^	50.03 ± 0.24^a,c^	12.12 ± 0.48^a,c^	36.30 ± 0.84^a,c^	37.03 ± 0.62^a,c^	47.65 ± 0.81^a,c^	12.55 ± 0.49^a,c^	42.76 ± 0.32^a,c^	41.80 ± 1.76^a,c^	44.23 ± 0.16^c^	13.91 ± 0.64^a,c^

a Significant difference compared with the control group (p < 0.05).

b Significant difference compared with group I (p < 0.05).

c Significant difference compared with group II (p < 0.05). ALP=Alkaline phosphatase, ALT=Alanine transaminase, AST=Aspartate transaminase, γGT=Gamma-glutamyl transpeptidase, SD=Standard deviation

**Figure 4:**
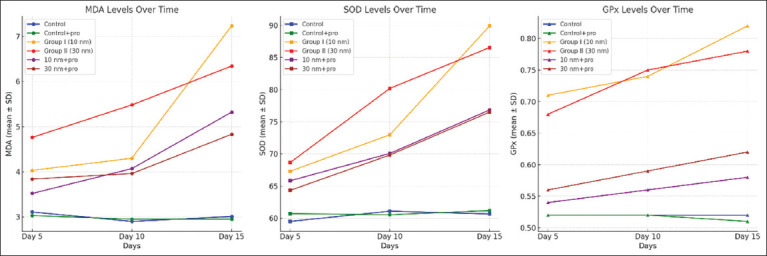
Trend of MDA, SOD, and GPx levels throughout the treatment period. The graphs show significant changes among all experimental groups over 5, 10, and 15 days. MDA= Malondialdehyde, SOD=*Superoxide dismutase*, GPx=Glutathione peroxides.

The blood MDA and SOD levels of the control and experimental groups are summarized in Figure 4 and Table 3. The GNP groups experienced higher amounts. However, after 5 days of treatment with two sizes of GNP + propolis, their levels had declined and were nearly identical to those of the control group (p < 0.01). The GPx activities of the various groups are shown in Table 3. The data indicated that compared with the control groups, the GNP group had considerably higher GPx levels. However, the elevation in the protective group was noticeably lower than that in the GNP group. The GPx levels in the 10 nm + propolis group decreased noticeably more than those in the 30 nm + propolis group. When examined for 5 days, the 10 nm + propolis group’s GPx levels were very similar to those of the control group.

**Table 3:** Serum level of MDA, SOD, and GPx (U/L) in different experimental groups at 5, 10, and 15 days (Mean ± SD).

Groups	5 days			10 days			15 days		
	MDA	SOD	GPx	MDA	SOD	GPx	MDA	SOD	GPx
Group 1 (Control)	3.11 ± 0.26	59.50 ± 1.23	0.52 ± 0.04	2.90 ± 0.11	61.10 ± 0.78	0.52 ± 0.03	3.01 ± 0.21	60.67 ± 1.03	0.52 ± 0.06
Group 2	3.03 ± 0.16	60.72 ± 1.32	0.52 ± 0.02	2.95 ± 0.12	60.55 ± 1.16	0.52 ± 0.01	2.95 ± 0.18	61.19 ± 1.41	0.51 ± 0.01
Group 3	4.03 ± 0.11^a^	67.30 ± 0.83^a^	0.71 ± 0.12 ^a^	4.30 ± 0.09^a^	72.98 ± 1.56^a^	0.74 ± 0.09^a^	7.23 ± 0.65^a^	89.91 ± 1.07^a^	0.82 ± 0.02^a^
Group 4	4.76 ± 0.18^a^	68.65 ± 1.07^a^	0.68 ± 0.05^a^	5.48 ± 0.11^a^	80.19 ± 1.19^a^	0.75 ± 0.03^a^	6.34 ± 0.25^a^	86.54 ± 0.45^a^	0.78 ± 0.03^a^
Group 5	3.52 ± 0.13^a,b^	65.82 ± 0.96^a,b^	0.54 ± 0.02^b^	4.07 ± 0.24^a,b^	70.08 ± 1.28^a,b^	0.56 ± 0.01^a,b^	5.32 ± 0.76^a,b^	76.83 ± 1.17^a,b^	0.58 ± 0.02^a,b^
Group 6	3.84 ± 0.48^a,c^	64.33 ± 2.19^a,c^	0.56 ± 0.01^a,c^	3.96 ± 0.13^a,c^	69.83 ± 1.17^a,c^	0.59 ± 0.01^a,c^	4.83 ± 0.29^a,c^	76.50 ± 1.17^a,c^	0.62 ± 0.02^a,c^

a Significant difference compared with the control group (p < 0.05).

b Significant difference compared with group I (p < 0.05).

c Significant difference compared with group II (p < 0.05). MDA=Malondialdehyde, SOD=Superoxide dismutase, GPx=Glutathione peroxides, SD=Standard deviation

**Figure 5:**
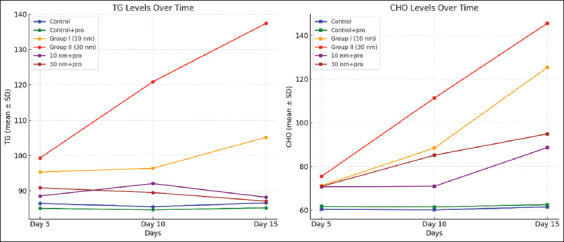
Trend of blood lipid (CHO and TG) profile throughout treatment. The graphs show significant changes among all experimental groups over 5, 10, and 15 days. CHO=Cholesterol, TG=Triglycerides.

Throughout the experiment, serum CHO and TG levels were substantially higher than expected after GNP treatment, as summarized in Figure 5 and Table 4. However, propolis therapy at various GNP sizes resulted in a noticeable decrease in serum propolis levels. On the other hand, the data revealed that rats administered oral propolis treatment had TG levels that were substantially identical to those of the control group, especially after 15 days of combination treatment (GNP with propolis).

**Table 4:** Serum level of TG (U/L) and CHO (U/L) in different experimental groups at 5, 10, and 15 days (Mean ± SD).

Groups	5 days		10 days		15 days	
		
	TG	CHO	TG	CHO	TG	CHO
Group 1 (Control)	86.45 ± 1.13	60.34 ± 0.83	85.55 ± 1.12	60.12 ± 0.25	86.61 ± 1.54	61.43 ± 1.33
Group 2	85.06 ± 1.40	61.70 ± 1.72	84.64 ± 0.72	61.49 ± 0.72	85.21 ± 1.93	62.50 ± 1.93
Group 3	95.34 ± 1.45^a^	71.23 ± 1.13^a^	96.41 ± 1.76^a^	88.43 ± 1.45^a^	105.16 ± 1.54^a^	125.32 ± 1.34^a^
Group 4	99.34 ± 2.32^a^	75.44 ± 1.43^a^	120.91 ± 2.65^a^	111.34 ± 1.23^a^	137.41 ± 1.43^a^	145.57 ± 1.26^a^
Group 5	88.55 ± 0.85^a,b^	70.71 ± 1.09^a^	92.08 ± 1.06^a,b^	70.90 ± 1.22^a,b^	88.19 ± 1.48^a,b^	88.66 ± 2.15^a,b^
Group 6	90.88 ± 1.19^a,c^	70.88 ± 1.28^a,c^	89.52 ± 1.08^a,c^	85.15 ± 1.31^a,c^	87.10 ± 0.55^c^	94.93 ± 1.34^a,c^

a Significant difference compared with the control group (p < 0.05).

b Significant difference compared with group I (p < 0.05).

c Significant difference compared with group II (p < 0.05). SD=Standard deviation, CHO=Cholesterol, TG=Triglycerides

The clinical signs of the rats were closely monitored during the study to assess the effects of GNPs and propolis. In general, no significant changes in behavior or health were observed in all groups. During the study, some fatalities were recorded across all groups, but the mortality rates remained within scientifically acceptable limits, ranging from 5% to15%. No unexpected or unexplained deaths occurred.

## DISCUSSION

Research has indicated that intravenous injection may interact with hepatocytes, liver sinusoidal endothelial cells, and Kupffer cells, leading to the hepatic deposition of GNPs [15]. However, these findings suggest that GNPs are hepatotoxic [16]. The current investigation found that GNP administration increased MDA, ALT, AST, ALP, and γGT. The increase in these parameters is a marker of oxidative stress, lipid peroxidation, and liver damage [17, 18]. According to Lala *et al*. [18], elevated levels of these biomarkers indicate a mixed cell damage pattern. MDA is the most reliable and straightforward metric for assessing the degree of lipid peroxidation under conditions of oxidative stress circumstances [19]. The most prevalent clinical symptom of liver damage is acute liver failure, which is characterized by significantly higher levels of liver function marker enzymes and abnormal liver function.

Propolis is an excellent hepatoprotective drug for treating acute liver failure. It inhibits lipid peroxidation while increasing tissue glutathione levels. It enhances antioxidant activity against toxicity by modulating the antioxidant enzyme system [12]. Antioxidants may protect cells against oxidative stress and damage from free radicals. Free radical scavenging may be necessary for propolis’s first mode of action; the second method might prevent the production of free radicals by inhibiting xanthine oxidase [20].

Propolis coadministration with GNP substantially reduced AST, ALT, ALP, γGT, and MDA levels compared with the GNP-treated group. This finding is consistent with Abdul-Hamid *et al*.’s [17] findings that propolis reduced the elevated activity of AST, ALT, ALP, and MDA in the serum of rats treated with ciprofloxacin. Furthermore, after 28 days of therapy, propetamphos (15 mg/kg b.w./day) was found to have negative effects on rats by increasing the levels of glucose and TG as well as the activities of AST, ALP, and ALT. The adverse effects of propetamphos were decreased in these animals after propolis administration (100 mg/kg b.w./day) [21].

SOD, another antioxidant enzyme, was measured and revealed a substantial increase, particularly during the 15 days in which the groups receiving GNPs were treated. The SOD enzyme lowers the damaging effects of ROS by converting toxic superoxide to hydrogen peroxide. In individuals with acute liver failure, elevated plasma SOD levels were linked to worsening of liver function. According to Tian *et al*. [22], elevated plasma SOD levels may be an adaptive reaction to a high level of systemic oxidative stress. Interestingly, rats administered propolis and GNPs daily showed a significant drop (p < 0.01) in their serum SOD levels. This study found that propolis extract significantly decreased SOD activity. This improvement may be crucial to the cellular defense against oxidative stress induced by propolis. This protective effect of propolis is in accordance with previous research [12, 23].

Oral GNP treatment in rats resulted in a high degree of oxidative stress in the liver. Antioxidant enzymes, including SOD and GPx, as well as non-enzymatic antioxidants like glutathione, exhibit enhanced activity in response to oxidative stress [24]. Rats administered propolis and GNPs had lower plasma levels of antioxidants, including SOD and GPx. Hassan *et al*. [25] reported that propolis prevents an increase in enzymes that are used as markers for liver function tests and the level of MDA, which indicates the degree of oxidative stress in liver tissues. Propolis also restored SOD and GPx enzymatic activities in the liver.

When a cell’s capacity to produce antioxidants is overwhelmed by the concentration of ROS produced, oxidative stress develops in that cell or tissue. Antioxidants can decrease oxidative damage by activating or inhibiting important enzyme systems. GNPs interact with the antioxidant systems of tissues and cells to produce free radicals. Naturally occurring living things produce ROS. The rate of ROS generation and enzymatic activity, such as GPx and SOD, is in balance under normal physiological conditions. Non-enzymatic antioxidants, including GPx and Vitamins A, E, and C, also control the rate of ROS generation. Oxidative stress is the result of excessive ROS formation and/or insufficient antioxidant defense, which causes the system to become imbalanced. Lipid peroxidation is the best measure of oxidative stress [19, 26]. These results suggest that aqueous propolis extract maintains GPx concentration reduction and prevents lipid peroxide production. Furthermore, tectochrysin, a significant propolis component, has antioxidant properties that may lessen MDA formation in rats that result from CCl4 poisoning [27, 28].

The serum lipid profile concentration increased noticeably due to GNPs. Significant increases in CHO and TG were observed in GNP-treated rats compared with the control group. With regard to the effect of orally administered propolis on lipid metabolism, the data revealed a hypolipidemic effect in all groups. Coadministration of propolis and GNPs led to reduced levels of TG; especially when treated for 15 days. It appears that TG levels returned to their normal levels, similar to the control, but the serum levels of total CHO were not restored to their normal levels as recorded in the control. Rats administered propolis had reduced liver TG and choline content and lowered hepatic TG synthesis rates [29]. Sajjad *et al*. [30] demonstrated that propolis kept the mice’s levels of CHO, TG, LDL, and HDL close to those of the control group. Propolis’s potential to treat dyslipidemia has been studied. The blood levels of LDL-C and total CHO in Kunming mice were reduced when HFD/STZ was administered; however, it had no effect on the serum TG level [31].

## CONCLUSION

Saudi propolis significantly reduced liver enzyme activities (AST, ALT, ALP, and GT), oxidative stress markers (MDA, SOD, and GPx), and lipid dysregulation (CHO and TG) in rats exposed to GNPs. Hepatoprotective effects were more pronounced in the 10 nm GNP + propolis group. This study incorporated a comprehensive biochemical evaluation of liver function, oxidative stress indicators, and lipid profiles, alongside the inclusion of multiple time points for temporal analysis and size-specific nanoparticle effects.

However, limitations include the study being restricted to male rats, reducing generalizability across sexes and species, the absence of exploration into specific mechanistic pathways like Nrf2 signaling and inflammatory markers, and the lack of long-term assessments on hepatic and systemic health. Future studies should investigate oxidative stress pathways and inflammatory markers for deeper mechanistic understanding, conduct dose-response analyses to determine optimal propolis dosages, expand research to female rats and other animal models, and explore long-term effects of propolis on liver and other organs. Translating these findings into preclinical and clinical studies could pave the way for therapeutic applications in humans.

This research highlights Saudi propolis as a promising natural antioxidant and hepatoprotective agent, providing a strong foundation for mitigating nanoparticle-induced liver toxicity and oxidative stress.

## DATA AVAILABILITY

The data generated during the study have been available in the manuscript.

## AUTHOR’S CONTRIBUTIONS

SAA: Designed and performed the study and drafted and revised the manuscript. The author has read and approved the final manuscript.
